# Association between the lactate-to-albumin ratio (LAR) index and risk of acute kidney injury in critically ill patients with sepsis: analysis of the MIMIC-IV database

**DOI:** 10.3389/fphys.2025.1469866

**Published:** 2025-02-19

**Authors:** Yiming Hua, Ning Ding, Huaizhi Jing, Yifei Xie, Hao Wu, Yue Wu, Beidi Lan

**Affiliations:** ^1^ Department of Cardiovascular Medicine, The First Affiliated Hospital of Xi’an Jiaotong University, Xi’an, Shaanxi, China; ^2^ Key Laboratory of Molecular Cardiology, Key Laboratory of Environment and Genes Related to Diseases, Ministry of Education, Xi’an Jiaotong University, Xi’an, Shaanxi, China; ^3^ Department of Cardiovascular Surgery, First Affiliated Hospital of Xi’an Jiaotong University, Xi’an, China

**Keywords:** lactate-to-albumin ratio, sepsis, acute kidney injury, MIMIC-IV database, ICU, intensive care unit

## Abstract

**Background:**

Lactate-to-albumin ratio (LAR) is an emergency predictive indicator of sepsis-related mortality. An elevated LAR is associated with poor outcomes in critically ill patients. However, its predictive value for acute kidney injury (AKI) in patients with sepsis remains unclear. Therefore, this study aimed to investigate the relationship between LAR and AKI in patients with sepsis.

**Methods:**

The study population was derived from the Medical Information Mart for Intensive Care-IV (2.0) database and stratified into quartiles based on the LAR. The primary endpoint was the occurrence of AKI. The secondary endpoints were the use of renal replacement therapy (RRT) and in-hospital mortality. Kaplan–Meier survival analysis and Cox proportional hazards models were used to assess the association between the LAR index and risk of AKI in patients with sepsis.

**Results:**

In this study, 5,222 patients with sepsis were included, of whom 3,029 were male (58%). Kaplan–Meier survival analysis demonstrated significant differences in the cumulative incidence of AKI and cumulative usage rate of RRT among patients with sepsis based on the quartiles of the LAR index. Additionally, Cox proportional hazards analysis adjusted for confounding factors showed a significant association between the LAR index and incidence of AKI in patients with sepsis.

**Conclusion:**

Our study indicated that a high LAR index can serve as an independent predictor of AKI in patients with sepsis.

## Introduction 

Sepsis is a complex disease caused by excessive activation of the immune system and an imbalance in the immune response to severe infections (Cecconi et al., 2018). Currently, it has a high global incidence and is one of the leading causes of death worldwide (Tiru et al., 2015). The incidence rate of sepsis is over 500 cases per 100,000 person-years (Fleischmann et al., 2016) and shows an increasing trend each year. Its mortality rate is as high as 30%, posing a significant burden on global healthcare (Cohen et al., 2015).

In addition, in sepsis, a dysregulated systemic inflammatory response can lead to microcirculatory dysfunction, resulting in the failure of vital organs. Inadequate renal perfusion, microvascular dysfunction, microcirculatory heterogeneity, inflammation, and metabolic reprogramming are common factors in sepsis that may contribute to acute kidney injury (AKI), thereby increasing the risk of mortality in sepsis patients (Poston and Koyner, 2019). Therefore, early identification of risk factors associated with sepsis is crucial for implementing preventive measures and preventing disease progression.

Albumin is a vital component of human bloodstream. It helps to maintain the balance of fluids inside and outside the blood vessels, thus sustaining normal osmotic pressure in the body (Fanali et al., 2012). Additionally, it binds to and transports various substances, including drugs, hormones, and trace elements. Furthermore, it plays a crucial role in maintaining the blood pH levels (Rothschild et al., 1988). Albumin is an important biomarker and is associated with various diseases, including cancer, rheumatoid arthritis, and obesity. Thus, it can be used clinically to treat multiple diseases (Fanali et al., 2012). Lactic acid is a byproduct of cellular metabolism that increases during tissue hypoxia and inadequate perfusion. Therefore, clinically, lactate can be used to assess the severity and prognosis of critically ill patients (such as those with trauma, infection, and sepsis). However, the use of lactate and albumin alone may be insufficient for better evaluation of certain complications. The lactate-to-albumin ratio (LAR) is a new indicator that has proven valuable for assessing mortality rates in patients with acute pancreatitis (Liu et al., 2022) and septic cardiac injury (Chen et al., 2023). Hypoalbuminemia in sepsis is caused by increased capillary permeability, increased distribution volume, and a reduced albumin production due to the increased formation of acute-phase proteins. The lactate-to-albumin ratio (LAR) can be considered an indicator of the heightened inflammatory response in sepsis. However, there are limited studies on the lactate-to-albumin ratio (LAR) in sepsis. One study have reported a correlation between the LAR index and the occurrence of AKI (Shi et al., 2024). It remains unclear whether LAR can assess mortality, acute kidney injury (AKI), and the need for renal replacement therapy in sepsis patients. Therefore, we aim to explore the prognostic value of the LAR index in critically ill patients with acute kidney injury. This study primarily investigates the relationship between LAR and the occurrence of AKI in patients with sepsis.

## Methods

### Study population

**FIGURE 1:**
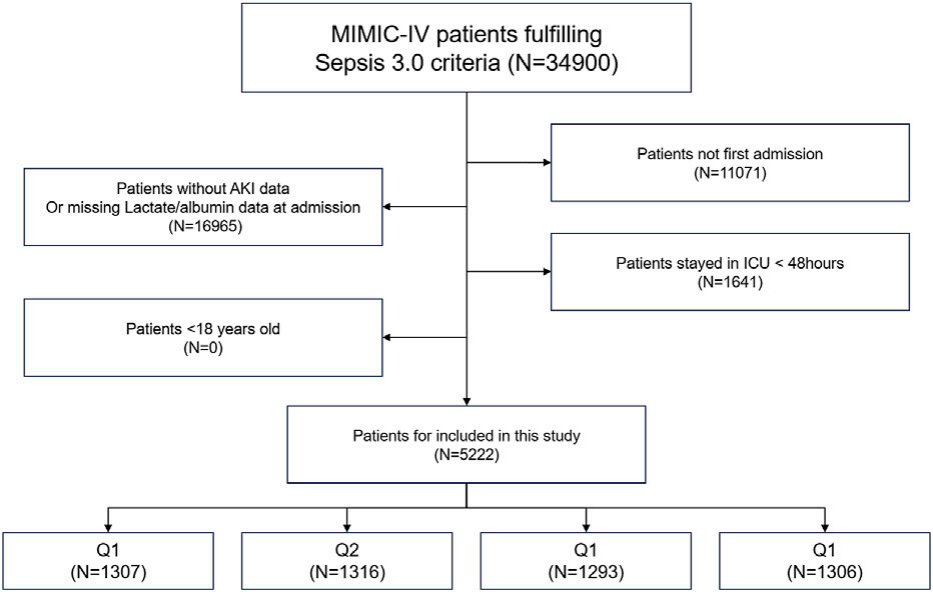
Overall flowchart of this study. MIMIC-IV: Medical Information Mart for Intensive Care IV.

This study was based on the freely accessible Medical Information Mart for Intensive Care-IV (MIMIC-IV) database, which records comprehensive information on all patients treated at the Beth Israel Deaconess Medical Center in Boston, Massachusetts, from 2008 to 2019. The data included vital signs, laboratory tests, medications, risk assessment indicators, and other relevant information. The first author (Yiming Hua) obtained official certification from the database and had research qualifications to access the data (ID: 52681986). To protect patient privacy, the database de-identifies personal information by replacing patient identities with random codes. In this study, the Sepsis-3 diagnostic criteria for sepsis include life-threatening organ dysfunction caused by infection, with a requirement for a SOFA score of ≥2. The SOFA score is used to assess the function of six organ systems (respiratory, coagulation, liver, cardiovascular, renal, and central nervous systems), with each organ scored based on the degree of dysfunction. The higher the SOFA score, the more severe the degree of organ failure (Singer et al., 2016). Exclusion criteria were: (1) non-first hospital admission, (2) lack of assessment data for AKI or LAR, (3) ICU stay <48 h, and (4) age <18 years (Figure 1).

### Data extraction

In this study, we used Structured Query Language to extract data from Navicat Premium software (version 12). All data were obtained from the MIMIC-IV database within the first 24 h of admission, including age, sex, BMI (Body Mass Index), heart rate (HR), systolic blood pressure (SBP), diastolic blood pressure (DBP), lymphocytes, neutrophils, platelets, white blood cells, red blood cells, hemoglobin, blood urea nitrogen (BUN), serum creatinine (SCR), glucose, anion gap, bicarbonate, calcium, chloride, sodium, potassium, international normalized ratio (INR), prothrombin time (PT), lactate, albumin, hypertension, myocardial infarction (MI), coronary artery disease (CAD), chronic heart failure (CHF), atrial fibrillation (AF), chronic kidney disease (CKD), liver disease, chronic obstructive pulmonary disease (COPD), diabetes, malignancy, percutaneous coronary intervention, and SOFA. The follow-up period began on the first day of hospital admission and ended on the date when AKI was first detected. The calculation formula for the LAR ratio is lactate (mmol/L)/albumin (g/dL). AKI is defined based on SCR and urine output in the first 48 h after ICU admission. All patients with sepsis in this study were evaluated for AKI. If a patient did not experience AKI in any hospital record during the follow-up period, they were considered to not have experienced AKI during the follow-up period. If a patient experienced AKI for the first time, the time of occurrence was recorded.

In our study, variables such as urine creatinine, CRP, and serum triglycerides with missing values exceeding 50% were excluded from the analysis. The rates of missing data used in this study are listed in Supplementary Figure S1. To handle missing data, we used the “missForest” package in R to impute data before fitting each model.

## Primary and secondary outcomes

The primary endpoint of this study was AKI incidence. AKI diagnosis criteria were based on the Kidney Disease: Improving Global Outcomes guidelines, with an increase in SCR to ≥1.5 times the baseline within 7 days or an increase of ≥0.3 mg per deciliter (mg/dL) in SCR within 24 h, or oliguria (Ostermann et al., 2020). The baseline SCR used in this study was within the first 24 h of admission. Renal replacement therapy (RRT) is an indirect indicator of AKI severity and was considered a secondary endpoint.

### Feature selection

**FIGURE 2:**
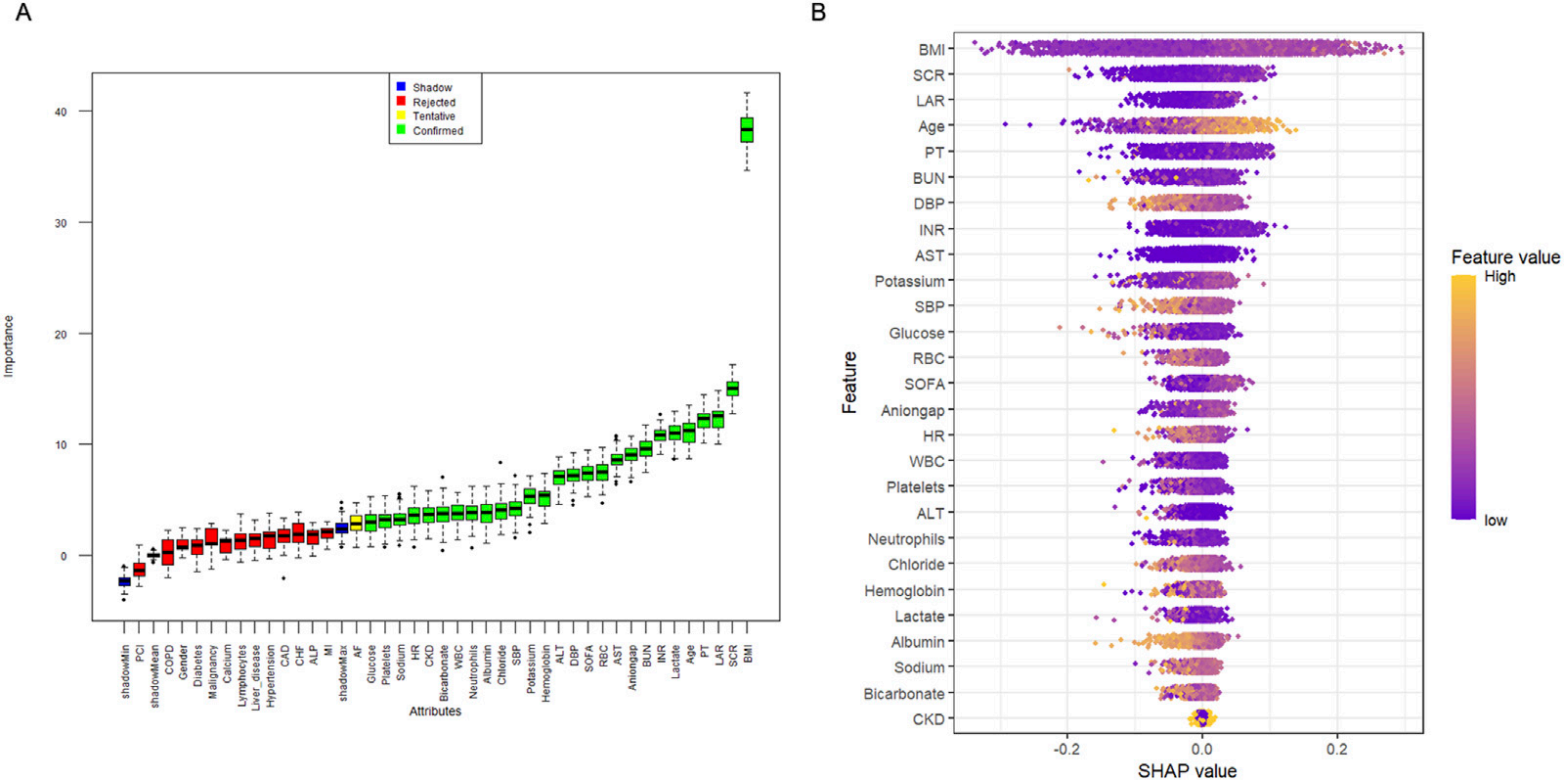
Application of Machine Learning in Feature Selection. **(A)** Feature selection for the relationship between various LAR index and AKI was analyzed using the Boruta algorithm. **(B)** Shapley Additive Explanations (SHAP) for the random forest model. A distribution of the impact of each feature on the model output. Each dot represents a patient in a row. The colors of the dots represent the feature values: yellow represents larger values and purple represents lower values.

Before investigating the impact of the LAR index on the occurrence of AKI in patients with sepsis, we employed a key method called the Boruta algorithm, which is a machine learning technique used to select feature variables and determine their importance in predictive models (Hoang et al., 2023). The Boruta algorithm generates shadow features based on data and identifies variables that have a significant impact on the outcomes (Figure 2A). Additionally, we fitted a random forest model for variable selection and used the SHapley Additive extension package (Figure 2B) to visualize the importance of the variables (Tseng et al., 2020). We included the selected variables in the subsequent analyses, which helped explain the results.

### Statistical analysis

For continuous variables, statistical descriptions were presented as means and standard deviations and between-group comparisons were conducted using either the Mann–Whitney U test or Student's t-test. Categorical variables were expressed as frequencies and percentages (%) and between-group comparisons were performed using Fisher’s exact test or Pearson’s chi-square test.

Kaplan–Meier survival analysis was used to assess the association between the LAR index and incidence of AKI and RRT usage in each group. Additionally, Cox proportional hazards model analysis was conducted to calculate the odds ratios (ORs) and their corresponding 95% confidence intervals (CIs) for the impact of the LAR index on the incidence of AKI between the different groups, adjusting for multiple variables that may affect the outcomes in the above analysis. No variable adjustments were made in Model 1. In Model 2, adjustments were made for age and BMI. In Model 3, based on prior literature (Liu et al., 2022) and the results of feature importance selection from the Boruta and random forest models (Yue et al., 2022) as shown in Figures 2A, B, variables included age, BMI, SCR, PT, LAR, lactate, INR, BUN, anion gap, SOFA, RBC, DBP, hemoglobin, potassium, SBP, chloride, bicarbonate, neutrophils, albumin, HR, sodium, CKD, platelets, and glucose. The impact of these additional factors was adjusted. In each model, the LAR index was included in both the continuous and categorical forms of adjustment. In all models, the lowest quartile of the LAR index was used as the baseline. Furthermore, restricted cubic splines (RCS) were applied to examine the relationship between LAR and outcome events, including AKI, renal replacement therapy (RRT).

Subgroup analyses were conducted to explore the consistency of the LAR index within different subgroups. These subgroups were defined based on sex (female vs. male), age (<65 vs. ≥65 years), BMI (<30 vs. ≥30 kg/m^2^), and the presence of diseases such as hypertension, CKD, liver disease, COPD, and malignancy.

The predictive value of the LAR index for the development of AKI in sepsis was evaluated using receiver operating characteristic (ROC) analysis (Supplementary Figure S2).

All data analyses were performed using R version 4.2.3. A two-sided P < 0.05 was considered statistically significant.

## Results

### Baseline characteristics

Table 1 presents the baseline characteristics of patients with sepsis divided into quartiles based on the LAR index (Q1: 0–0.35; Q2: 0.35–0.5; Q3: 0.5–0.79; and Q4: >0.79). The averages for these four groups were 0.27 ± 0.05, 0.42 ± 0.04, 0.63 ± 0.08, and 1.49 ± 0.90, respectively. The incidence rates of AKI in the four groups were 77.4%, 82.0%, 82.1%, and 87.8%, respectively (P < 0.0001). Among patients with sepsis in the Q4 group, relatively young age, high HR, and low SBP and DBP were observed. Laboratory tests revealed low levels of lymphocytes, neutrophils, RBC, hemoglobin, bicarbonate, calcium, and albumin in the Q4 group. However, relatively high levels of BUN, SCR, glucose, anion gap, potassium, INR, PT, and lactate were observed in the Q4 group. Additionally, the Q4 group had a lower prevalence of comorbidities, such as hypertension, MI, CAD, CHF, and COPD, but a higher prevalence of liver disease and malignancy, along with higher SOFA scores. (all P < 0.05).

**TABLE 1:** Baseline characteristics according to LAR index quartiles.

	Q1 (0,0.35]	Q2 (0.35,0.50]	Q3 (0.50,0.79]	Q4 (0.79,10]	Pvalue
	(N = 1,307)	(N = 1,316)	(N = 1,293)	(N = 1,306)	
Demographic					
Age	62.77 ± 17.28	64.14 ± 16.49	64.10 ± 16.87	62.47 ± 16.79	0.014
Gender					0.264
Male	752 (57.5)	770 (58.5)	774 (59.9)	733 (56.1)	
Female	555 (42.5)	546 (41.5)	519 (40.1)	573 (43.9)	
BMI	29.90 ± 8.60	30.09 ± 9.03	29.76 ± 8.62	29.60 ± 7.81	0.624
Vital Signs					
HR (beats/min)	85.21 ± 16.63	87.89 ± 16.80	91.30 ± 17.31	96.69 ± 18.36	<0.001
SBP (mmHg)	117.94 ± 14.98	116.10 ± 15.47	113.94 ± 14.95	109.75 ± 13.54	<0.001
DBP (mmHg)	62.16 ± 10.60	62.68 ± 10.71	61.90 ± 10.41	61.15 ± 10.24	0.002
Laboratory tests					
Lymphocytes,%	12.57 ± 11.49	10.24 ± 9.59	10.66 ± 10.22	10.04 ± 10.31	<0.001
Neutrophils,%	79.00 ± 13.95	80.91 ± 12.44	78.67 ± 15.09	78.02 ± 15.15	<0.001
Platelets (K/uL)	244.09 ± 123.55	233.29 ± 118.90	220.49 ± 123.33	207.28 ± 135.26	<0.001
WBC (K/uL)	12.84 ± 8.91	13.72 ± 7.60	14.30 ± 8.23	15.00 ± 8.85	<0.001
RBC (K/uL)	3.62 ± 0.73	3.66 ± 0.73	3.60 ± 0.77	3.47 ± 0.75	<0.001
Hemoglobin (g/dL)	11.63 ± 2.30	11.87 ± 2.30	11.74 ± 2.36	11.59 ± 2.34	0.012
BUN (mg/dL)	32.57 ± 27.49	34.88 ± 27.18	36.76 ± 27.57	38.98 ± 26.79	<0.001
SCR (mg/dL)	1.87 ± 2.04	1.92 ± 1.87	1.95 ± 1.69	2.23 ± 1.82	<0.001
Glucose (mg/dL)	177.91 ± 104.73	198.17 ± 116.47	210.89 ± 128.29	221.17 ± 132.22	<0.001
Aniongap (mEq/L)	17.06 ± 4.85	17.77 ± 5.02	18.65 ± 5.58	21.72 ± 6.99	<0.001
Bicarbonate (mEq/L)	25.18 ± 4.93	24.21 ± 4.78	23.42 ± 4.54	21.82 ± 4.52	<0.001
Calcium (mg/dL)	8.65 ± 0.85	8.56 ± 0.95	8.55 ± 0.98	8.54 ± 1.39	0.009
Chloride (mEq/L)	106.68 ± 6.81	106.58 ± 6.80	107.08 ± 7.50	107.13 ± 7.76	0.125
Sodium (mEq/L)	140.74 ± 5.07	140.56 ± 5.62	140.69 ± 6.30	140.88 ± 6.56	0.619
Potassium (mEq/L)	4.69 ± 0.93	4.72 ± 0.90	4.80 ± 0.99	4.95 ± 1.00	<0.001
INR	1.53 ± 1.19	1.66 ± 1.24	1.84 ± 1.36	2.32 ± 1.82	<0.001
PT	16.48 ± 10.62	18.18 ± 12.75	19.85 ± 13.40	25.19 ± 19.55	<0.001
Lactate (mmol/L)	0.92 ± 0.22	1.34 ± 0.29	1.84 ± 0.44	3.63 ± 2.03	<0.001
Albumin (g/dL)	3.44 ± 0.59	3.16 ± 0.62	2.93 ± 0.64	2.55 ± 0.67	<0.001
LAR	0.27 ± 0.05	0.42 ± 0.04	0.63 ± 0.08	1.49 ± 0.90	<0.001
Comorbidities,n(%)					
Hypertension	530 (40.6)	483 (36.7)	496 (38.4)	419 (32.1)	<0.001
MI	239 (18.3)	284 (21.6)	221 (17.1)	219 (16.8)	0.005
CAD	339 (25.9)	367 (27.9)	284 (22.0)	265 (20.3)	<0.001
CHF	414 (31.7)	461 (35.0)	377 (29.2)	339 (26.0)	<0.001
AF	356 (27.2)	399 (30.3)	387 (29.9)	363 (27.8)	0.214
CKD	294 (22.5)	298 (22.6)	266 (20.6)	251 (19.2)	0.097
Liver_disease	164 (12.5)	266 (20.2)	349 (27.0)	538 (41.2)	<0.001
COPD	417 (31.9)	386 (29.3)	309 (23.9)	273 (20.9)	<0.001
Diabetes	389 (29.8)	405 (30.8)	388 (30.0)	384 (29.4)	0.889
Malignancy	122 (9.3)	149 (11.3)	190 (14.7)	235 (18.0)	<0.001
PCI	12 (0.9)	20 (1.5)	11 (0.9)	10 (0.8)	0.201
SOFA	3.59 ± 1.93	4.10 ± 2.26	4.40 ± 2.42	5.11 ± 2.96	<0.001
AKI,n(%)	1,012 (77.4)	1,079 (82.0)	1,061 (82.1)	1,147 (87.8)	<0.001

BMI: Body Mass Index, HR: heart rate, SBP: systolic blood pressure, DBP: diastolic blood pressure, WBC: white blood cells, RBC: red blood cells, BUN: blood urea nitrogen, SCR: serum creatinine, INR: international normalized ratio, PT: prothrombin time, MI: myocardial infarction, CAD: coronary artery disease, CHF: chronic heart failure, AF: atrial fibrillation, CKD: chronic kidney disease, COPD: Chronic obstructive pulmonary disease, diabetes, PCI: percutaneous coronary intervention, SOFA: sequential organ failure assessment.

### LAR index, incidence of AKI, RRT usage, and in-hospital mortality

**FIGURE 3:**
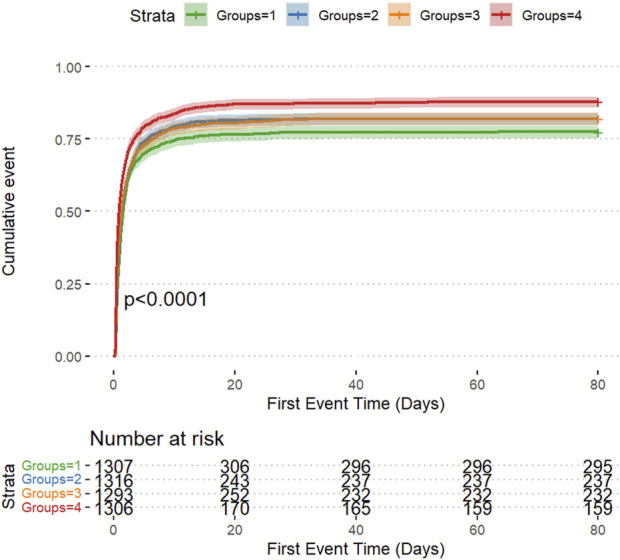
The cumulative event incidence curves for incidence of AKI.

**FIGURE 4:**
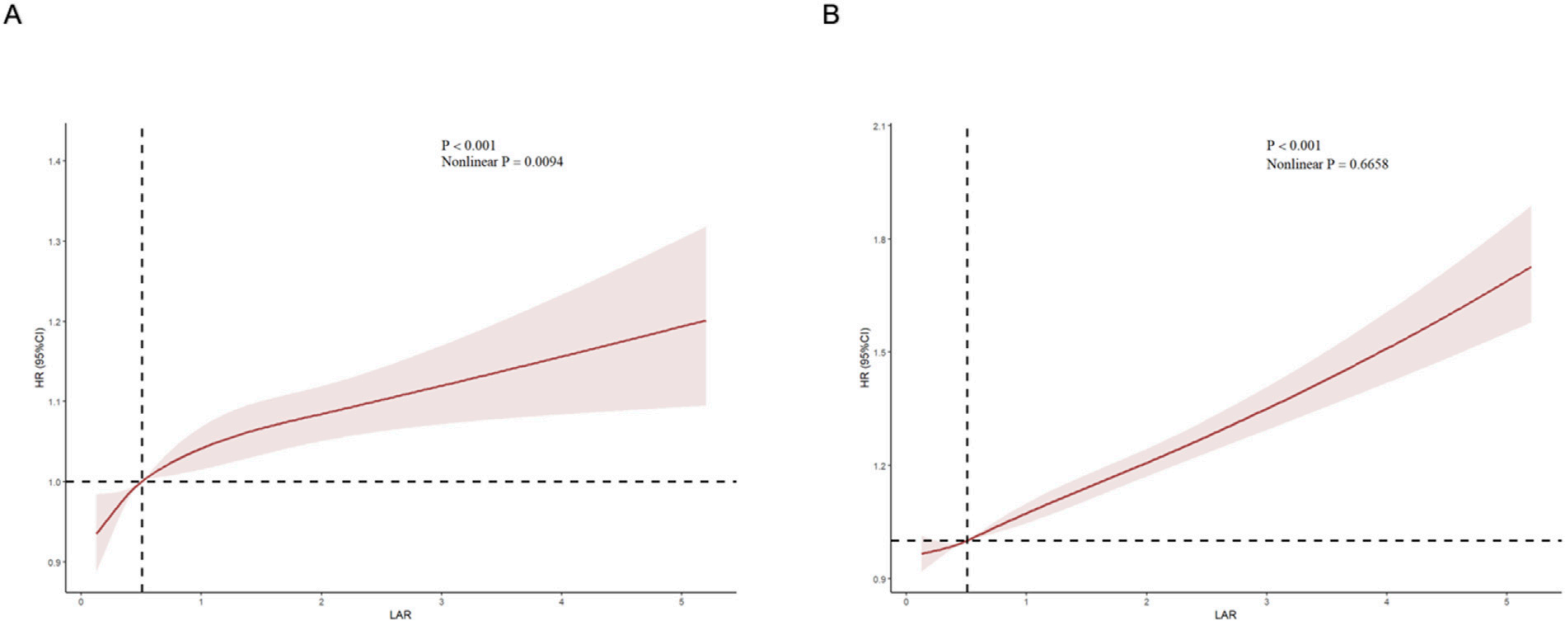
Restricted cubic spline (RCS) showing the relationship between LAR and outcome indicators. **(A)** RCS showed the correlation between LAR and AKI **(B)** RCS showed the correlation between LAR and RRT.

**FIGURE 5:**
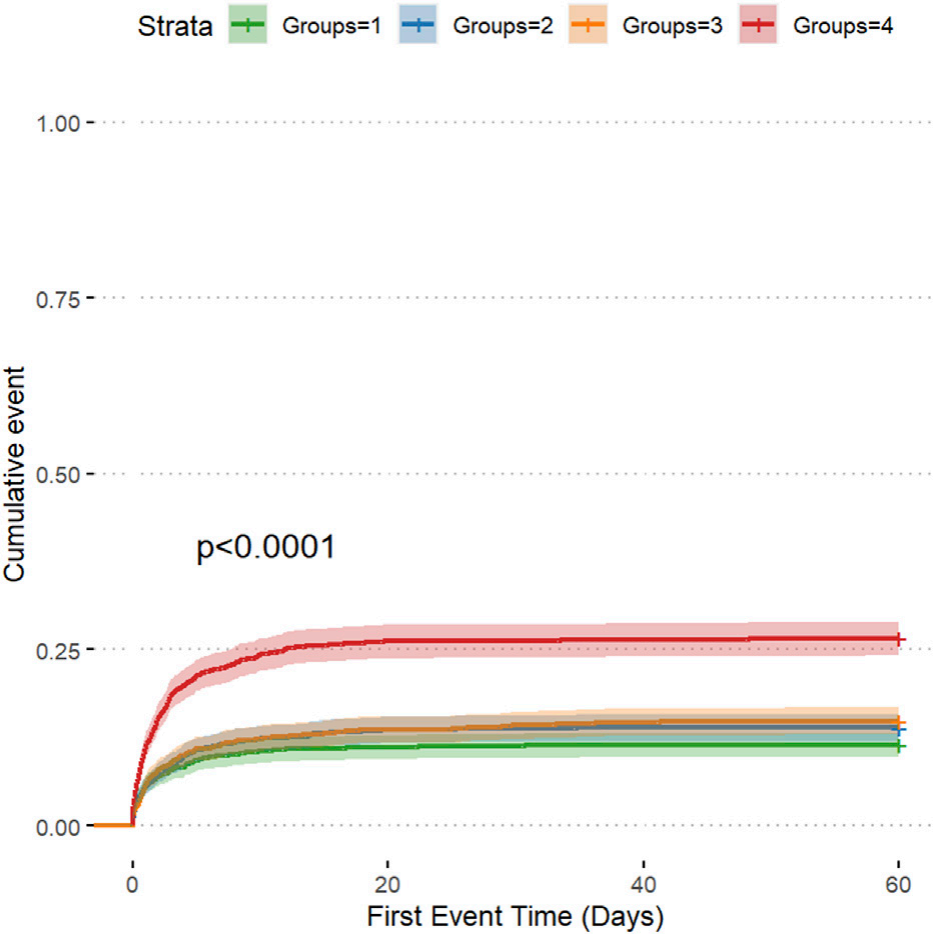
The cumulative event incidence curves for use of RRT.

The cumulative risk curve displays the cumulative event incidence curve for AKI based on the LAR index quartiles. Throughout the follow-up period, there were significant differences in the incidence of AKI among the groups (P < 0.0001) (Figure 3). Figure 4 illustrates the restricted cubic splines regression model, highlighting the dose-response relationship between LAR and the risks of AKI incidence and RRT usage (nonlinear p = 0.0094, nonlinear p < 0.001, and nonlinear p = 0.6658, all p < 0.001). The restricted cubic spline regression models for AKI incidence and RRT usage demonstrate a significant positive correlation with LAR scores, indicating that the risks of AKI occurrence and RRT usage increase with higher LAR. The cumulative risk curves indicated that the highest quartile group, Q4, had the highest cumulative RRT usage, which was statistically significant (Figure 5). Additionally, the use of RRT and risk of in-hospital mortality consistently increased with an increasing LAR index, with all p-values below 0.05 (Table 2).

**TABLE 2:** Outcome events.

	Q1 (0,0.35]	Q2 (0.35,0.50]	Q3 (0.50,0.79]	Q4 (0.79,10]	Pvalue
	(N = 1,307)	(N = 1,316)	(N = 1,293)	(N = 1,306)	
RRT	150 (11.5)	183 (13.9)	192 (14.8)	348 (26.6)	<0.001
Los_hospital	14.90 ± 14.45	15.90 ± 13.78	16.89 ± 15.73	17.60 ± 16.45	<0.001
Los_icu	7.86 ± 7.65	8.03 ± 7.24	8.14 ± 7.57	8.59 ± 7.74	0.093
Death at hospital	181 (13.8)	237 (18.0)	309 (23.9)	487 (37.3)	<0.001

RRT: renal replacement therapy, Los_hospital: length of stay in hospital, Los_icu: length of stay in ICU.

Cox proportional hazards model analysis indicated that the LAR index was independently associated with an increased incidence of AKI (OR, 1.163 [95% CI 1.015–1.333; P = 0.029]). In the highest quartile group Q4, the LAR index showed a significant association with the risk of AKI in both the unadjusted (Q1 vs. Q2: HR, 1.099 [95% CI 1.008–1.198] P = 0.032; Q3: HR, 1.082 [95% CI 0.989–1.182] P = 0.083; Q4: HR, 1.198 [95% CI 1.070–1.341] P = 0.001) and fully adjusted models (Q1 vs. Q2: HR, 1.029 [95% CI 0.940–1.127] P = 0.527; Q3: HR, 1.029 [95% CI 0.930–1.138] P = 0.574; Q4: HR, 1.163 [95% CI 1.015–1.333] P = 0.029), indicating statistical significance (Table 3).

**TABLE 3:** Cox proportional hazard ratios (HR) for AKI incidence.

Categories	Model 1		Model 2		Model 3	
	HR (95%CI)	Pvalue	HR (95%CI)	Pvalue	HR (95%CI)	Pvalue
AKI incidence						
LAR as continuous	1.177 [95%CI 1.1078–1.250]	<0.001	1.159 [95%CI 1.091–1.231]	<0.001	1.051 [95%CI 0.909–1.216]	0.499
Quartile^a^						
Q1	Ref.		Ref.		Ref.	
Q2	1.099 [95%CI 1.008–1.198]	0.032	1.073 [95%CI 0.984–1.170]	0.100	1.029 [95CI% 0.940–1.127]	0.527
Q3	1.082 [95%CI 0.989–1.182]	0.083	1.092 [95%CI 0.998–1.170]	0.049	1.029 [95%CI 0.930–1.138]	0.574
Q4	1.198 [95%CI 1.070–1.341]	0.001	1.246 [95%CI 1.112–1.395]	<0.001	1.163 [95%CI 1.015–1.333]	0.029

Model 1 was unadjusted.

Model 2 was adjusted for Age and BMI.

Model 3 Age, BMI, SCR, PT, LAR, Lactate, INR, BUN, Anion gap, SOFA, RBC, DBP, Hemoglobin, Potassium, SBP, Chloride, Bicarbonate, Neutrophils, Albumin, HR, Sodium, CKD, Platelets, and Glucose.

### Subgroup analysis

**FIGURE 6:**
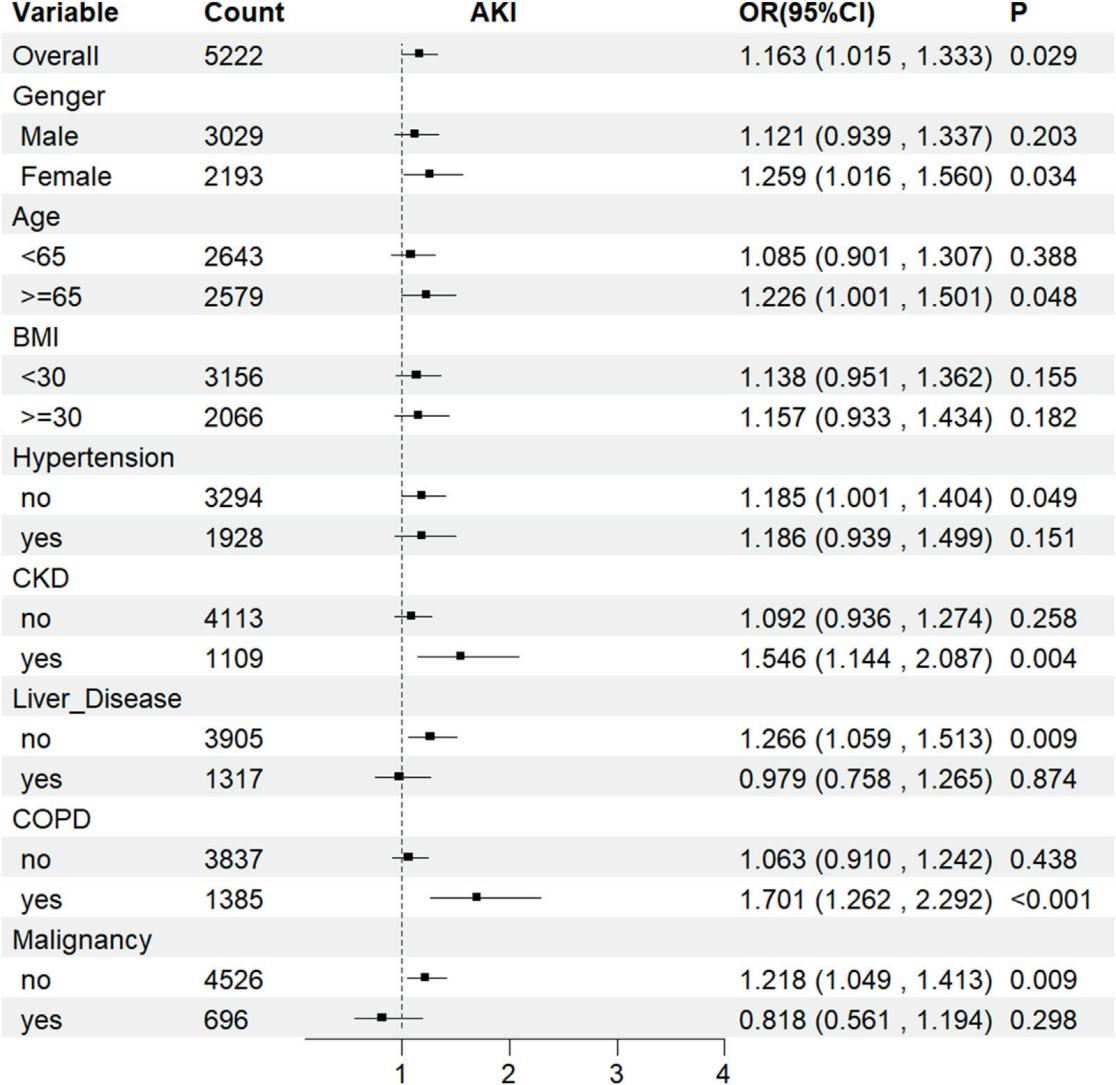
Subgroup analyses for the association of LAR index with AKI. OR: odds ratio, CI: confidence interval.

The LAR index was associated with AKI incidence across multiple subgroups based on sex, age, hypertension, CKD, COPD, liver disease, and malignancy (Figure 6). Furthermore, the LAR index showed a statistically significant association with an increased risk of AKI occurrence in these indicators: female sex (HR [95% CI] 1.259 [1.016–1.560]), age≥65 years (HR [95% CI] 1.226 [1.001–1.501]), absence of hypertension (HR [95% CI] 1.185 [1.001–1.404]), presence of CKD (HR [95% CI] 1.546 [1.144–2.087]), absence of liver disease (HR [95% CI] 1.266 [1.059–1.513]), presence of COPD (HR [95% CI] 1.701 [1.262–2.292]), and absence of malignancy (HR [95% CI] 1.218 [1.049–1.413]) (all P < 0.05).

## Discussion

This study is the first to confirm that the LAR is an important predictive indicator and a reliable biomarker for predicting the occurrence of AKI in patients with sepsis. Notably, this association remained statistically significant even after adjusting for potential confounding factors. The analysis revealed that a higher LAR index was associated with a higher incidence of AKI and RRT usage. Despite adherence to clinical treatment guidelines, sepsis remains a common and severe condition, primarily originating from various sources of severe infections such as pneumonia, abdominal infections, and urinary tract infections, and is associated with higher mortality rates, thereby posing an increasing burden on global public health (Rhodes et al., 2017). Bagshaw et al. (2007) found that nearly half of patients with AKI had sepsis in the ICU. In our study, sepsis was the sole influencing factor in 43% of patients. The only multicenter epidemiological study comparing septic and non-septic AKI conducted by Neveu et al. (1996) found that 46% of AKI patients had a septic origin. Both studies consistently showed that the mortality rate of AKI patients with sepsis was higher than that of patients with sepsis who did not develop AKI. Consequently, it is crucial to discover a novel biomarker that can identify patients with sepsis at risk of developing AKI, with the aim of enhancing their outcomes.

This corresponds with the results of previous studies suggesting that the LAR is associated with ICU mortality and short-term all-cause mortality in patients with sepsis, demonstrating its significant clinical application value (Yoo et al., 2024). Lactic acid as a biomarker reflects the degree of tissue hypoperfusion in patients with sepsis, with elevated levels indicating a poor prognosis of sepsis (Trzeciak et al., 2007), which can lead to AKI. However, by including albumin in the assessment, the LAR provides a more comprehensive measure of physiological disturbances and the body’s response to inflammation in patients with sepsis, which are key changes during the progression of sepsis. Additionally, the dual measurement index of the LAR is superior to traditional indicators, such as C-reactive protein and procalcitonin, which are single-factor indicators that cannot reflect metabolic disturbances in patients with sepsis. The LAR index is strongly linked to in-hospital cardiac arrest (IHCA). Individuals exhibiting elevated LAR scores demonstrate lower survival rates post-IHCA compared to those with reduced scores. Furthermore, patients who regain spontaneous circulation after IHCA show a diminished likelihood of attaining favorable neurological outcomes (Haschemi et al., 2023). Intracerebral hemorrhage (ICH), a particularly severe form of stroke, also exhibits high fatality rates. In patients suffering from ICH, a higher LAR index correlates with an increased probability of mortality both in the hospital and within ICU settings (Wu et al., 2023).

However, this study also has limitations. First, this is an observational study, and despite using rigorous and correct statistical analysis methods, we cannot establish a definitive causal relationship between LAR index and outcome indicators. Second, this was a single-center study and despite adjustments for various confounding factors and subgroup analyses, potential data bias may still exist. Third, due to the inherent limitations of the MIMIC database, many other factors, such as the severity of AKI, etiology of sepsis, and socioeconomic status of the included patients, were not considered, which could have affected the results. Fourth, our focus was solely on the predictive value of the baseline LAR index for AKI prognosis in patients with sepsis, without considering changes in the LAR index, which is also a limitation of the MIMIC database.

## Conclusion

In this study, an elevated LAR index was closely associated with an increased incidence of AKI in patients with sepsis. Our findings suggest that LAR can serve as an independent factor for assessing the risk of AKI in patients with sepsis and can guide subsequent interventions. Nevertheless, we recommend that large-scale prospective studies be conducted to validate our results.

## Data Availability

The original contributions presented in the study are included in the article/Supplementary Material, further inquiries can be directed to the corresponding author.

## Abbreviations

LAR, lactate-to-albumin ratio; AKI, acute kidney injury; RRT, renal replacement therapy; MIMIC-IV database, Medical Information Mart for Intensive Care-IV database; BMI, Body Mass Index; HR, heart rate; SBP, systolic blood pressure; DBP, diastolic blood pressure; BUN, blood urea nitrogen; SCR, serum creatinine; INR, international normalized ratio; PT, prothrombin time; MI, myocardial infarction; CAD, coronary artery disease; CHF, chronic heart failure; AF, atrial fibrillation; CKD, chronic kidney disease; COPD, chronic obstructive pulmonary disease; PCI, percutaneous coronary intervention; SOFA, sequential organ failure assessment; OR, odds ratios; CIs, confidence intervals; IHCA, in-hospital cardiac arrest.
