# Supplementary figures and images for: Association between the lactate-to-albumin ratio (LAR) index and risk of acute kidney injury in critically ill patients with sepsis: analysis of the MIMIC-IV database
Source: Front Physiol. 2025 Feb 19;16:1469866. doi: 10.3389/fphys.2025.1469866 (PMC11879934; doi:10.3389/fphys.2025.1469866)

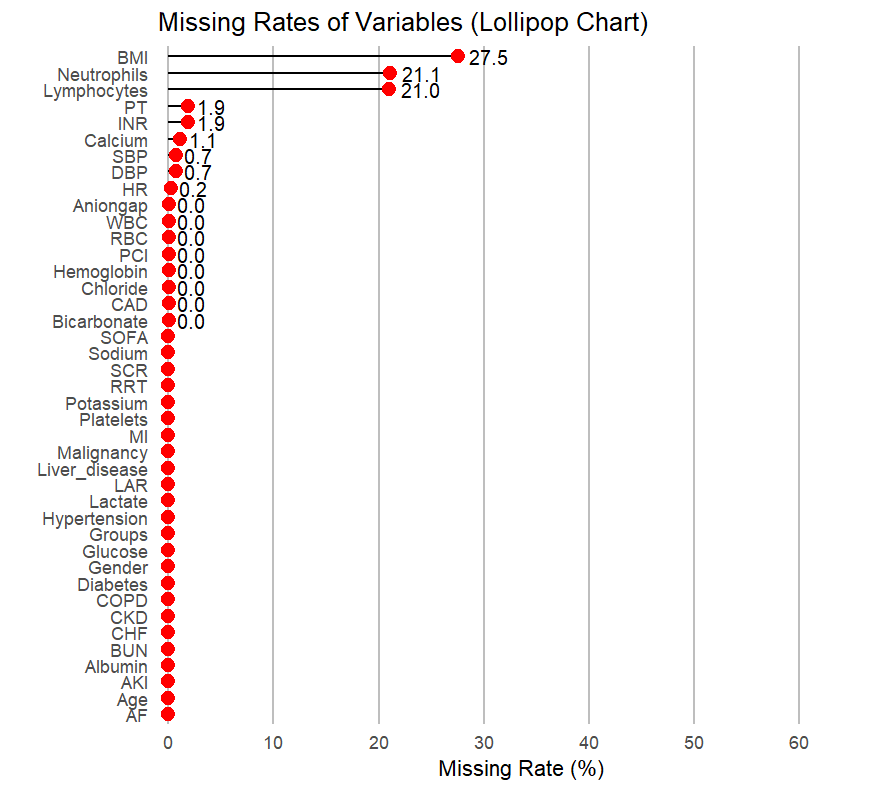

Supplement: Supplementary file 1 [file Image1.tiff]

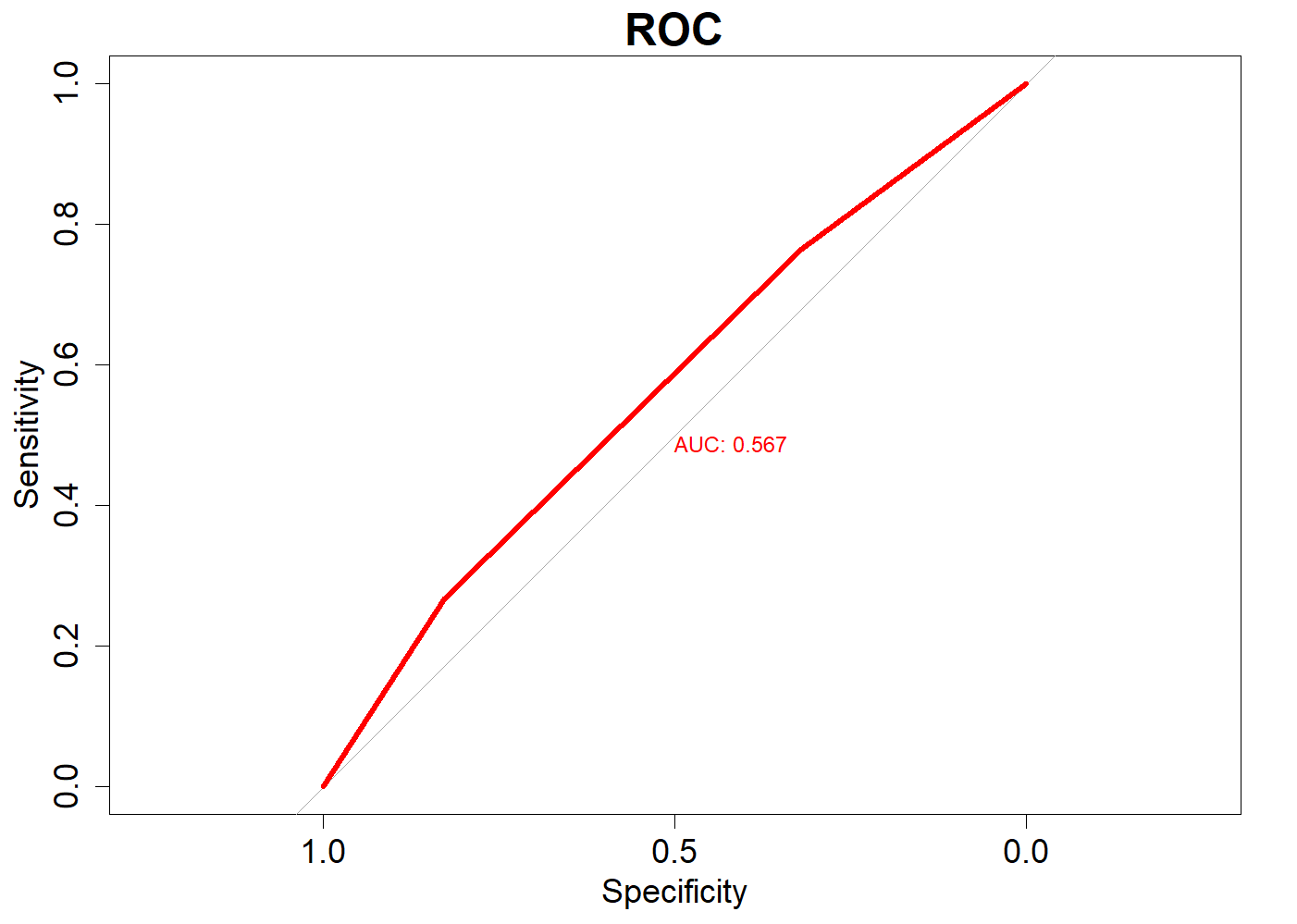

Supplement: Supplementary file 2 [file Image2.tiff]
